# Evaluation of Food Security in North China Based on Food Production Level

**DOI:** 10.3390/foods13142189

**Published:** 2024-07-11

**Authors:** Junqi Cheng, Shuyan Yin

**Affiliations:** School of Geography and Tourism, Shaanxi Normal University, Xi’an 710119, China; chengjq789@163.com

**Keywords:** factor analysis, food production influencing factors, food security evaluation, spatial econometric modelling, North China

## Abstract

This paper focuses on county-level grain production and food security in North China; selects 17 indicators from both climatic conditions and human activities; applies yield fluctuation coefficients, spatial econometric modelling, the random forest method, and factor analysis to study the characteristics of grain production in North China and the influencing factors; and evaluates the situation of food security in North China based on grain production capacity. The following results were obtained: (1) The spatial and temporal changes in grain production located in North China from 2000 to 2020 are obvious. The grain output in North China from 2000 to 2020 maintains fluctuating growth at a rate of 0.38 × 10^11^ kg/10a. The east and south are the key areas for grain production in North China. Grain output was relatively stable except for 2003. with the cold spots of grain production being mainly in the northwestern area and the hot spots in the central and southern areas. (2) The changes in grain production in North China from 2000 to 2020 were less affected by climate and mainly influenced by human activity indicators. (3) As time progresses, the area of food shortage zones decreases in size, becoming evenly distributed and dispersed from the initial concentration in northern Hebei and most of Shanxi; the change in the supply–demand equilibrium zones is not obvious; and the area of surplus grain zones increases markedly in size, with a tendency to expand from the south and centre of the study area to the west and north. The grain production capacity of counties in the northwest and north is generally low, and even counties located in surplus grain areas have potential food security risks. However, in the east and south, due to their high grain production capacity, the per capita grain supply situation may be alleviated even in counties located in grain shortage areas. This study can deepen the understanding of the characteristics of food production in North China and enrich the research on food security. Analyses of factors influencing food production will improve a deeper understanding of food security. Food security evaluation based on food production capacity will contribute to a more precise and comprehensive understanding of the food security pattern in North China.

## 1. Introduction

Food is an indispensable material basis for human survival and is equally essential for other human activities. Food security plays an important role in maintaining social progress and human well-being, and the issue of food security has once again attracted great attention from all walks of life as a result of the ongoing occurrence of public security incidents at home and abroad, which have had a very serious impact on food production and supply. The Global Food Crisis Report, jointly published by the United Nations World Food Programme and multiple agencies, shows that more than 25 million people will face food security problems in the second half of 2020 in East Africa alone, and that the number of people suffering from severe hunger will reach 265 million globally, an increase of 96% compared to 2019 [1,2]. The global outbreak of the COVID-19 epidemic has led many countries around the world to ban or reduce food exports, blocking the food supply chain, increasing the risk of global food security, and increasing the crisis of local conflicts [3]. Food security is the cornerstone of China’s social development [4]. With 7% of the world’s arable land feeding one-fifth of the world’s population, China has made remarkable achievements in food production. However, in the process of urbanization, the relationship between human and land has become increasingly tense [5], and the contradiction between agricultural development and the ecological environment has become prominent [6]. In addition, barriers to factor mobility between urban and rural areas and shifts in farmers’ livelihoods [7,8] pose significant threats to food production [9]. Therefore, it is necessary to analyse the spatial and temporal characteristics of food security and risk patterns, which is of great significance to ensure food security and promote sustainable economic development in China.

China is a large country in terms of population as well as food production and consumption, and its food security has received great attention from the government. Studies on food security have focused on the topics of food production and supply [10,11], food policy [12,13,14], and key drivers [15,16]. Climate factors and human intervention are the two main factors affecting food production, and there is insufficient research on the comprehensive impact of multiple factors. China’s food security still faces many challenges. The global spread of COVID-19, the rise of trade protectionism, the instability of the international geopolitical pattern, and the increased risks and uncertainties faced by the global food market have not only greatly increased the external market risks and pressures faced by China’s food security, but also brought unprecedented shocks and challenges to the domestic food supply and demand balance [17]. For this reason, based on the domestic and international environment in the new period, it is necessary to rethink the key issues and countermeasures involved in food security.

Previous research scales have mainly focused on large-scale regions such as global and national, and there is a lack of overall research on a long time series for a specific geographic region. North China is one of the main grain-producing areas in China. In the past 30 years, 45% of the increase in national grain production originated from the North China Plain, which plays an important role in guaranteeing national food security [18]. North China is also an area of high concentration of industries, population, and towns in northern China. Since the 1990s, due to rapid urbanization and economic development, a large amount of farmland has been encroached upon, bringing enormous pressure to crop production and food security in the region. In order to better reveal the food security issues facing North China, it is urgent to quantitatively analyse the influencing factors of the food security pattern. Although there are more research results on food security in North China, it can still be improved upon in the following aspects: firstly, how does the location of food production in North China change? Secondly, what kinds of impacts do climatic factors and human interventions have on food production? Finally, what are the characteristics of the level of food security in North China? In view of this, this paper adopts highly relevant climate and socio-economic data on grain production and food production from 2000 to 2020 in North China, and makes use of the fluctuation coefficient method, spatial autocorrelation, spatial lag, and factor analysis to conduct more systematic analyses of the grain production pattern and food security pattern in North China. The main research objectives of this paper are to (1) analyse the current situation of grain production in North China; (2) reveal the influencing factors of grain production in North China; (3) conduct a comprehensive evaluation of food security in North China, in order to provide practical suggestions for China’s food policy development from a more macro perspective.

## 2. Data and Methods

### 2.1. Study Area

**Figure 1:**
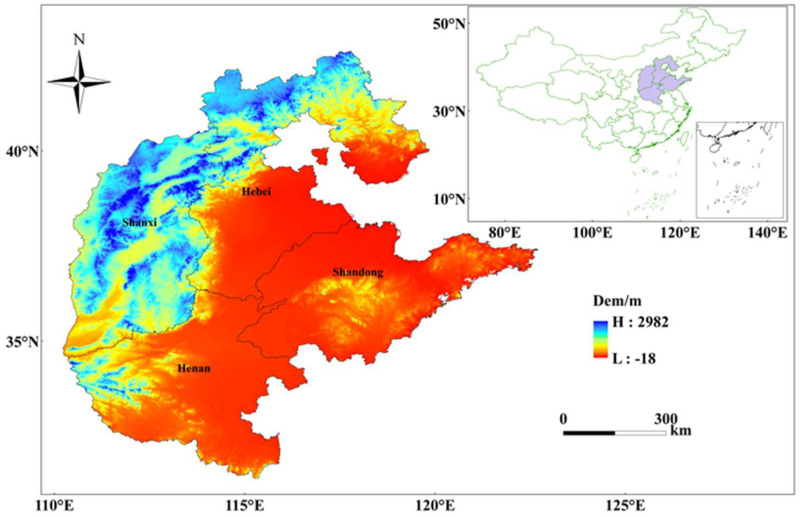
Location map of the study area.

Different papers in the literature have slightly different definitions of North China [19]. In this paper, the study area includes Shanxi, Hebei, Henan, and Shandong provinces based on the unity, integrity, and data completeness of the study area (Figure 1). North China has a temperate semi-humid continental climate with an average annual precipitation of 400–800 mm, mainly concentrated in summer. North China is located between the three major river basins of the Yellow River, the Huaihe River, and the Haihe River, with vegetation types dominated by woodlands and grasslands, and large plains in the central and eastern parts of the region, which are the main crop areas in China.

The North China Plain is an important grain, cotton, and oil production base in China with fertile soil, deep soil layer, good lighting conditions, sufficient heat, and convenient irrigation. Grain crops mainly include wheat, corn, rice, millet, sorghum, and potatoes, while economic crops mainly include cotton, peanuts, sesame, tobacco, and soybeans. Mainly based on a double cropping system, it is a typical winter wheat–summer corn planting system. However, summer floods, spring droughts, severe soil salinization, and poor surface drainage severely limit the development of its agriculture.

Hebei, Shandong, and Henan provinces have historically been China’s top grain-producing areas and major grain redeployers, while Shanxi Province is China’s grain supply–demand balance area, and grain production in these provinces is of great significance in guaranteeing China’s food security.

### 2.2. Data

Taking the county level as the unit of study, this paper collects data on county-level food production and population size from 2000–2020 from the statistical yearbooks of Shaanxi, Hebei, Henan, and Shandong provinces, edited by the corresponding provincial municipal statistical bureaus and the provincial survey offices of the National Bureau of Statistics (NBS). These statistical yearbooks were downloaded from the China National Knowledge Infrastructure (CNKI) online database (https://navi.cnki.net/knavi/yearbooks/index?uniplatform=NZKPT, accessed on 1 July 2023) after careful quality control and error checking processes. Therefore, the source of data is reliable. Missing data for individual years in some counties were supplemented using the moving average method [20].

The main climate dataset used is the daily value dataset, which mainly contains indicators such as temperature, precipitation, and sunshine hours. The dataset is released by the National Meteorological Science Data Centre of China (https://data.cma.cn, accessed on 1 August 2023), and the day-by-day precipitation, temperature, and sunshine data are selected for the period of 2000–2020, which covers 78 meteorological stations in the selected study area. RClimDex (1.0) was used to strictly check and control the data information of the selected stations, mainly including the time consistency test, the elimination of outliers and erroneous values, the extreme value test, etc. The very few missing data were interpolated by linear interpolation to ensure the completeness and consistency of the data, and the meteorological data of the stations were processed by the difference, and their spatial distributions were obtained by superimposing the DEM, which eliminated the non-data sources such as elevation, slope, etc. The data were also processed by the DEM. The influence of non-climatic factors such as altitude and slope was eliminated. In order to more accurately reveal the influence of climatic factors on crop yields in the study area, the average temperature; precipitation; the average temperature and precipitation of the growing season (March to October); sunshine hours; ≥0 °C cumulative temperature; ≥10 °C cumulative temperature; the number of consecutive drought days; the growing period of the crop; and the average daily temperature difference, which are closely related to agricultural development, were selected as the meteorological inputs of the measurement model. Climatic data were extracted at the county level for the study area using the ArcGIS zonal statistics tool and county administrative maps.

### 2.3. Methods

#### 2.3.1. Indicator Construction

From the aspects of climate change and human activities, we selected 17 indicators that are closely related to food production, including average annual temperature (Tem), annual precipitation (Pre), average temperature during the growing season (Tem3-10), precipitation during the growing season (Pre3-10), hours of sunshine (Ssd), ≥0 °C cumulative temperature (Ta ≥ 0), ≥10 °C cumulative temperature (Ta ≥ 10), consecutive drought days (Cdd), growing period of the crop (Gsl), average daily temperature difference (Dtr), total population of the region (Pop), Gross Domestic Product (GDP), sown area of grains (Gsa), pure fertiliser application (Fer), total power of agricultural machinery (Mac), and rural electricity consumption (Elec). These were used to determine the factors affecting food production.

#### 2.3.2. Analysis of Spatial Patterns

The spatial measurement method can effectively identify the spatial distribution of a certain geographic element, and the GIS platform provides a hot spot analysis tool based on the Getis–Ord Gi* statistical index. By calculating the Z score between each patch, it can directly reflect the clustering of high-value areas (hot spots) and low-value areas (cold spots) in space, and the higher the Z value, the more obvious the clustering of hot spots [21]. The formula is as follows:

(1) $$G_{i}^{*}=\frac{\sum_{j}\left(w_{ij}\times x_{j}\right)}{\sum_{j}x_{j}}$$

where Gi* is the local spatial autocorrelation index, x_j_ is the crop production in county j, and w_ij_ is the spatial adjacency matrix between county i and county j. Based on the Z scores and *p*-values of the Getis–Ord Gi* index, the spatial agglomeration of grain production in North China was classified into five classes at statistical significance levels of 0.01 and 0.05: Ggrade I hot spot (high value, *p* < 0.01), Grade II hot spot (high value, *p* < 0.05), Grade I cold spot (low value, *p* < 0.01), and Grade II cold spot (low value, *p* < 0.05).

#### 2.3.3. Spatial Measurement Models

Considering that the data used in the exploration of factors affecting grain production include both time series and cross-sectional data, using only traditional OLS estimation that ignores spatial effects may lead to bias in the model setting process, resulting in biased regression results [15]. However, spatial econometric models embed spatial interaction effects and focus on the interaction effects between variables due to spatial dependence and spatial spillover. Therefore, a spatial panel model [13] is used to examine the influencing factors of grain production. Spatial panel econometric models include three types: spatial lag model (SLM), spatial error model (SEM), and spatial Durbin model (SDM) [22] with the following formulae:

Spatial lag model:

(2) Y=μWY+Xβ+ε

Spatial error model:

(3) Y=Xβ+εε=δWε+λ

Spatial Durbin model:

(4) $$Y=\mu WY+X\beta +W\bar{X}r+\varepsilon$$

In the expressions, *Y* is the dependent variable, *μ* is the endogenous interaction effect coefficient reflecting the degree of spatial overflow, *β* is the regression coefficient, $\bar{X}$ represents the explanatory variable, *r* is the coefficient of the explanatory variable, and *ε* is the random error term.

This article mainly uses two Lagrangian multipliers (LM) in the form of LMerr and LMlag statistics, as well as robust R-LMerr and R-LMlag tests to determine the final model selection [23].

#### 2.3.4. Random Forest

On the basis of spatial econometric analysis, the random forest method is used to further evaluate the contribution rate of climate change and human intervention to grain production in North China, in order to enhance the accuracy of research results. Random forest is a relatively new machine learning model. Random forest uses a random method to build a forest; the forest contains many decision trees, as does the classifier. Each decision tree is independent, and its output category is determined by the output category of a single tree. As a commonly used algorithm, the random forest has a lot of advantages, and a lot of research has already shown that the combination of classifiers is better than a single classifier when it comes to the data analysed by the random forest model. The process of classifying and discriminating the data uses multiple classification trees, which classify the data. At the same time, it is able to calculate the score of the importance of each variable and evaluate the role played by each variable [24]. The specific algorithm is as follows:

- (1) Algorithm for classification trees

The sample ${D^{\prime }}_{1m}$ is divided into two ensembles using the optimal cut-off point a. The optimal cut-off point for indicator m is determined by the Gini coefficient. Firstly, the minimum Gini coefficient is calculated for each indicator until there are no indicators to choose from; secondly, the Gini coefficients are ranked, and finally a classification tree is generated.

(5) $$Gini\left({D^{\prime }}_{1m},m\right)=min\left(\frac{\left|{D^{\prime }}_{{1ma}^{-}}\right|}{\left|{D^{\prime }}_{1m}\right|}*\left(1-\sum_{j=1}^{5}\frac{\left|{D^{\prime }}_{{1mja}^{-}}\right|}{\left|{D^{\prime }}_{{1ma}^{-}}\right|}\right)+\frac{\left|{D^{\prime }}_{{1ma}^{+}}\right|}{\left|{D^{\prime }}_{1m}\right|}*\left(1-\sum_{j=1}^{5}\frac{\left|{D^{\prime }}_{{1mja}^{+}}\right|}{\left|{D^{\prime }}_{{1ma}^{+}}\right|}\right)\right)$$

(6) $${D^{\prime }}_{1m}=\left\{\begin{array}{c} {D^{\prime }}_{{1ma}^{-}},x_{mj}^{k}\leq a\\ {D^{\prime }}_{{1ma}^{+}},x_{mj}^{k}>a \end{array}\right.$$

In the expression, $Gini\left({D^{\prime }}_{1m},m\right)$ represents the Gini coefficient, ${D^{\prime }}_{1m}$ represents the set, m represents the optimal cut-off metric, a represents the cut-off point, ${D^{\prime }}_{{1ma}^{-}}$ represents the left set of the a cut-off on m, and ${D^{\prime }}_{{1ma}^{+}}$ represents the right set of the a cut-off on *m*.

- (2) Calculation of importance

Using the classification tree, the Gini coefficient of each indicator on the full classification tree is calculated, which in turn determines the importance of the indicator.

(7) $$VIM\left(m\right)=\frac{\sum_{n_{1}=1}^{n_{1}}Gini\left({D^{\prime }}_{n_{1}m},\left\{n_{1},v_{1}\right\}\right)}{\sum_{n_{1}=1}^{n_{1}}\sum_{m=1}^{18}Gini\left({D^{\prime }}_{n_{1}m},\left\{n_{1},v_{1}\right\}\right)}$$

In the expression, VIM(m) stands for importance, m stands for metrics, $n_{1}$ stands for classification tree, ${D^{\prime }}_{n_{1}m}$ stands for training set, $\left\{n_{1},v_{1}\right\}$ stands for coefficient parameter, $n_{1}$ stands for number of classification tree, and $v_{1}$ stands for number of random metrics.

#### 2.3.5. Factor Analysis

Factor analysis is an objective and quantitative method to determine the weights of indicators, and the core idea of factor analysis is dimensionality reduction, which converts many indicators into a few indicators containing various types of information composite indicators called explanatory factors, and then the explanatory factors are used to reflect the relationship between the factors and variables [25]. In this paper, the factor analysis method is used to determine the dominant factors affecting food production in North China, and then evaluate its food security. The process of factor analysis method is as follows:

- (1) Setting the original system matrix

(8) $$X=\left[\begin{array}{cccc} x_{11} & x_{12} & \ldots & x_{1n}\\ x_{21} & x_{22} & \ldots & x_{2n}\\ \ldots & \ldots & \ldots & \ldots \\ x_{t1} & x_{t2} & \ldots & x_{tn} \end{array}\right]$$

where *n* is the number of indicators and *t* represents the year; since the indicators have different units, it is necessary to avoid negative impacts due to differences in the units of the indicators, and it is necessary to carry out an outline process as follows:

(9) $$Z_{ij}=\frac{x_{ij}-\bar{x}}{\sqrt{var\left(x_{j}\right)}}i=1,2\cdots t;j=1,2\cdots n$$

- (2) Extracting the common factor

(10) $$R={\left(r_{jk}\right)}_{n\times n}\left[\begin{array}{cccc} r_{11} & r_{12} & \cdots & r_{1n}\\ r_{21} & r_{22} & \cdots & r_{2n}\\ \cdots & \cdots & \cdots & \cdots \\ r_{n1} & r_{n2} & \cdots & r_{nn} \end{array}\right]j=1,2,\cdots ,n;k=1,2,\cdots ,n$$

included among these $r_{jk}=\frac{1}{n-1}\sum_{i=1}^{n}\left(z_{ij}-{\bar{z}}_{j}\right)\left(z_{ik}-{\bar{z}}_{k}\right)$, formulae $r_{ii}=1,r_{jk}=r_{kj},i=1,2,\cdots ,t;j=1,2,\cdots ,n;k=1,2,\cdots ,p$, *λ* is the eigenvalue; *μ* is the eigenvector; *R* is the matrix; *λ* and *μ* can be obtained by the eigenequation $\left|\lambda I-R\right|=0$; and the common factor can be obtained by the magnitude of the cumulative contribution, and the eigenvectors, to create the loading matrix:

(11) $$A=\left[\begin{array}{cccc} a_{11} & a_{12} & \cdots & a_{1t}\\ a_{21} & a_{22} & \cdots & a_{2t}\\ \cdots & \cdots & \cdots & \cdots \\ a_{n2} & a_{n2} & \cdots & a_{nt} \end{array}\right]=\left[\begin{array}{cccc} \mu_{11}\sqrt{\lambda_{1}} & \mu_{12}\sqrt{\lambda_{2}} & \cdots & \mu_{1q}\sqrt{\lambda_{q}}\\ \mu_{21}\sqrt{\lambda_{1}} & \mu_{21}\sqrt{\lambda_{2}} & \cdots & \mu_{2q}\sqrt{\lambda_{q}}\\ \cdots & \cdots & \cdots & \cdots \\ \mu_{n1}\sqrt{\lambda_{1}} & \mu_{n2}\sqrt{\lambda_{2}} & \cdots & \mu_{nq}\sqrt{\lambda_{q}} \end{array}\right]$$

In the above equation, $a_{ij}$ represents the load matrix, $\mu_{ij}$ represents the eigenvectors, and $\lambda_{i}$ represents the eigenvalues, which are related as follows:

(12) $$a_{ij}=\mu_{ij}\sqrt{\lambda_{j}}i=1,2,\cdots ,n;j=1,2,\cdots ,t$$

- (3) Factor Rotation

Extracted public factors: if not very typical of each variable, the public factors need to display appropriate factor rotation to obtain a more satisfactory main factor. The rotation method has two types of rotation: orthogonal rotation and oblique rotation; this paper adopts the most commonly used maximum variance orthogonal rotation method for the factor rotation, so that, in the factor loadings in the factor loading matrix of the factor loadings squared value of the factor loadings in the direction of the two directions of 0 and 1, the larger loadings are larger and the smaller loadings are smaller [26].

- (4) Factor scores

The scores for the public factors are calculated for each indicator using the following formula:

(13) $$\left\{\begin{array}{c} f_{1}=a_{11}x_{1}+a_{21}x_{2}+\cdots +a_{n1}x_{n}\\ f_{2}=a_{12}x_{1}+a_{22}x_{2}+\cdots +a_{n2}x_{n}\\ \cdots \\ f_{k}=a_{1k}x_{1}+a_{2k}x_{2}+\cdots +a_{nk}x_{n} \end{array}\right.$$

The expression $F_{k}$ denotes the interpretation factor.

- (5) Composite score

The composite score F is calculated as follows:

(14) $$F=\sum_{i=1}^{q}a_{i}f_{i}i=1,2,\cdots ,q$$

where a represents the contribution of each explanatory factor.

## 3. Results

### 3.1. Characteristics of Grain Production

**Figure 2:**
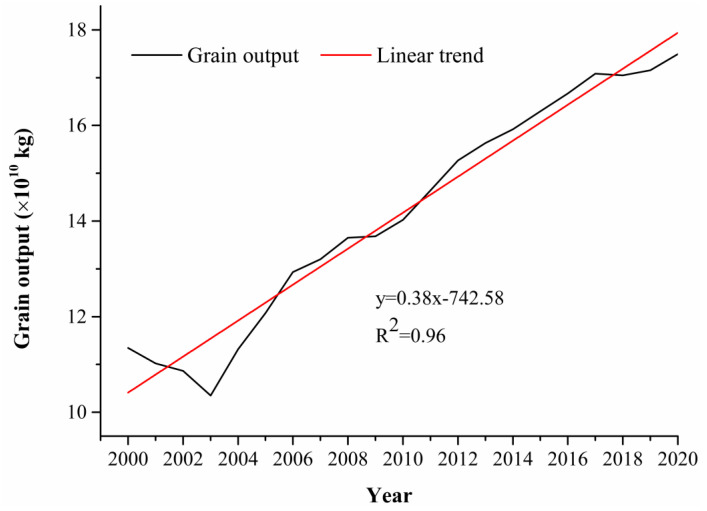
Changes in grain production in North China from 2000–2020.

Since 2000, grain production in North China has generally maintained fluctuating growth (Figure 2). It increased from 11.34 × 10^10^ kg in 2000 to 17.49 × 10^10^ kg in 2020, with a total increase of 61.49 × 10^10^ kg and an average annual growth rate of 2.58%. Figure 2 shows that after 2000, grain production in North China entered an obvious stagnation stage. Combined with relevant research results, it can be found that this phenomenon is mainly caused by a combination of factors such as the reduction of grain planting area, insufficient labour supply, backward production technology level, farmers’ low motivation for production and natural disasters (flood and drought) [25]. In stages, the first stage from 2000 to 2003 is a rapid decline stage; the average annual growth rate of −2.19%, grain production in 2003 reached the lowest point of 10.35 × 10^10^ kg. The second phase, 2003–2017, was a growth period with an average annual growth rate of 4.34%. The third phase, 2017–2019, was a stable phase with little change in grain production. Thereafter, it entered into a growth phase again.

**Figure 3:**
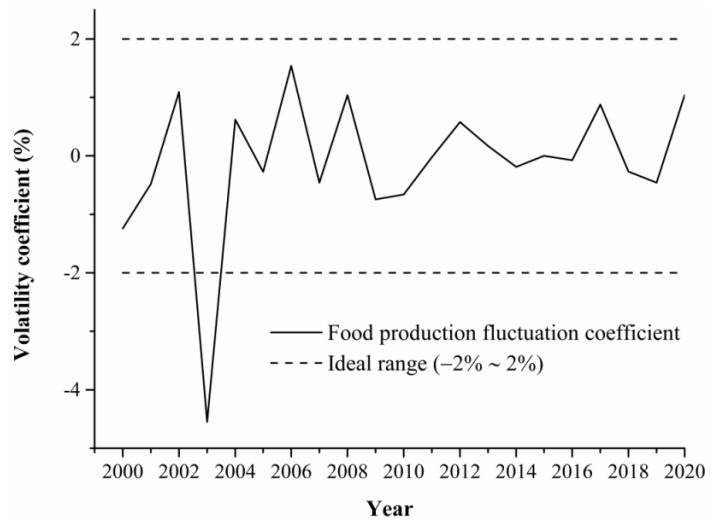
Characteristics of grain output fluctuations in North China from 2000–2020.

Food production is more stable. Grain production has been exposed to natural risks for a long time, and there are some inter-annual variations in grain production, and it is generally considered that the ideal interval for the grain fluctuation coefficient is −2% to 2% [24], and grain production is considered to be unstable when it exceeds this limit. The grain production fluctuation coefficient of North China in the period of 2000–2020 was the largest in 2006, and the smallest in 2003, with an average fluctuation coefficient of −0.12%. Except for 2003, when it exceeded the ideal range, grain production was relatively stable in other years, with a fluctuation coefficient of ±2% (Figure 3).

### 3.2. Spatial Patterns of Food Production

**Figure 4:**
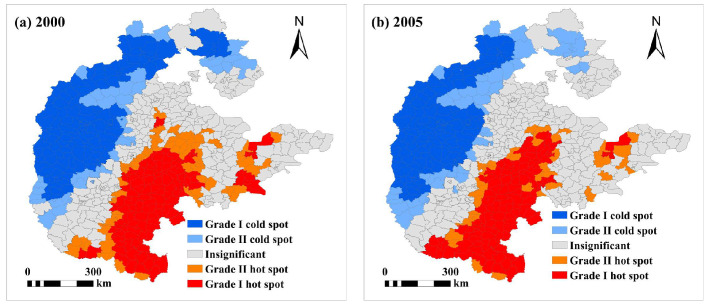

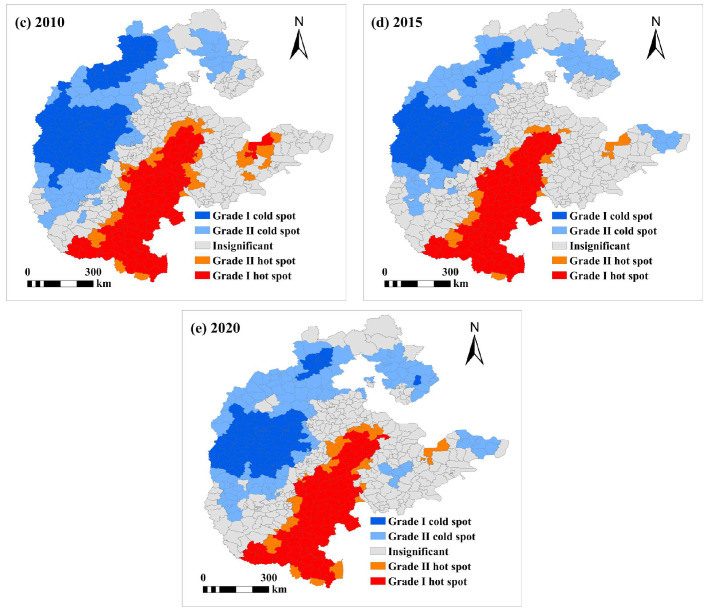
Hot spots and cold spots of crop production in the. North China at the county level from 2000–2020.

The hot and cold spots of food production changed less obviously, with cold spots mainly in the northwest and hot spots in the centre and south (Figure 4). Between 2000 and 2020, the hot and cold spots of food production changed, with the area of both hot spots and cold spots shrinking, with the area of Grade Ⅰ cold spots shrinking most significantly. Before 2015, Grade Ⅰ hot spots were also distributed in the eastern part of the plains, and no Grade Ⅰ hot spots have been found in the area since 2015, with the area of Grade Ⅰ hot spots first increasing and then decreasing. Before 2015, Grade Ⅰ hot spots were also distributed in the eastern part of the plains, and since 2015, no Grade Ⅰ hot spots have been found in the region. The area of Grade Ⅰ hot spots increases and then decreases. Grade Ⅱ hot spots are distributed around Grade Ⅰ hot spots, and the area decreases significantly. The spatial distribution pattern of Grade Ⅰ cold spots has changed significantly, with the area in the northwestern part of the region shrinking significantly. Grade Ⅱ cold spots were not distributed in the east before 2015 and appeared in the east after 2015.

### 3.3. Influence Factors

#### 3.3.1. Model Testing and Selection

According to the analysis process of the spatial econometric model, LM and R-LM tests were done on grain production and influencing factors in North China, and the test results are shown in Table 1.

It can be seen that in 2000, 2005, 2010, and 2015, LMlag, R-LMlag, LMerr, and R-LMerr passed the significance test of 0.01, indicating that all three models in these three years are suitable for spatial econometric analyses in this paper. In 2020, LMlag and R-LMlag passed the significance test of 0.1, while LMerr and R-LMerr did not pass any level of significance test. The test results indicate that the 2020 spatial lag model is more suitable for spatial econometric analysis in this paper.

Combining the analyses of the test results for each of the above years, the SLM model is the most reasonable choice for spatial econometric analyses in this paper.

#### 3.3.2. Analysis of Results

Over the past 20 years, each influencing factor has had a significant impact on grain production in different years. Grain sown area, fertilizer application, and total power of agricultural machinery in North China passed the significance test for grain production from 2000 to 2020, and all of them were significantly positive, indicating that these three factors made outstanding contributions to promoting grain production from 2000 to 2020; precipitation promoted grain production in 2000–2005; sunshine hours promoted grain production in 2000–2005; the impact of population on grain production was strengthening, and promoted grain production in 2020; the significance of other factors on grain production in North China was unstable (Table 2).

### 3.4. Contribution of Climate Change and Human Activities to Food Production

Since the factors that cause changes in grain yield are intricate and complex, and different factors affect grain yield to different degrees, how to screen out the main factors affecting grain yield is an issue well worth in-depth investigation.

In this paper, we use the random forest method to conduct the importance analysis of the original sequence of grain production in North China over the years with each influencing factor, calculate the importance of each influencing factor, and further analyse the main factors affecting grain production in North China. Looking at the statistics of the importance of each factor over the years (Table 3), the average contribution of climate change to food production over the years was 6.52%, with the largest contribution being sunshine hours, and the importance of climate factors in descending order are as follows: Ssd > Cdd > Dtr > Gsl > Pre > Ta ≥ 10 > Tem3-10 > Ta ≥ 0 > Pre3-10 > Tem. The contribution of human activities to food production reaches 93.48%, of which the importance of grain sown area ranks first, with a contribution of 69.62%, followed by fertilizer application and total power of agricultural machinery, and the importance of human activity indicators, in descending order, are as follows: Gsa > Fer > Mac > Pop > Elec > GDP, which shows that the changes in grain production in North China from 2000 to 2020 are mainly caused by human activities.

### 3.5. Food Security Evaluation

#### 3.5.1. Selection of Indicators

In order to evaluate the food security situation in North China and fully consider the actual situation of food production in North China, a food security evaluation system is constructed on the basis of the previous study; combining the relevant research results, the indicator system for food security evaluation is constructed, and the indicator system constructed in this paper is shown in Table 4.

#### 3.5.2. Food Security Classification in the Context of Food Production Capacities

This paper constructs an evaluation system for food security in North China, using factor analysis to calculate the comprehensive score of food production capacity of county units in North China in 2000, 2005, 2010, 2015, and 2020, and the classification of food security level is based on the height of the score of food production capacity, with the average value as the measure. A high score implies a high level of food production and a good food security situation; a low score implies a relatively low level of food production and a potential risk to food security.

**Figure 5:**
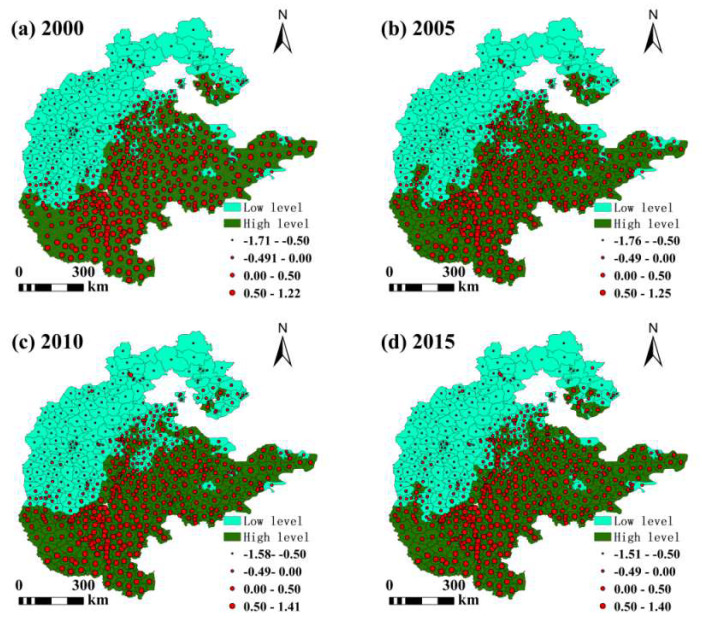

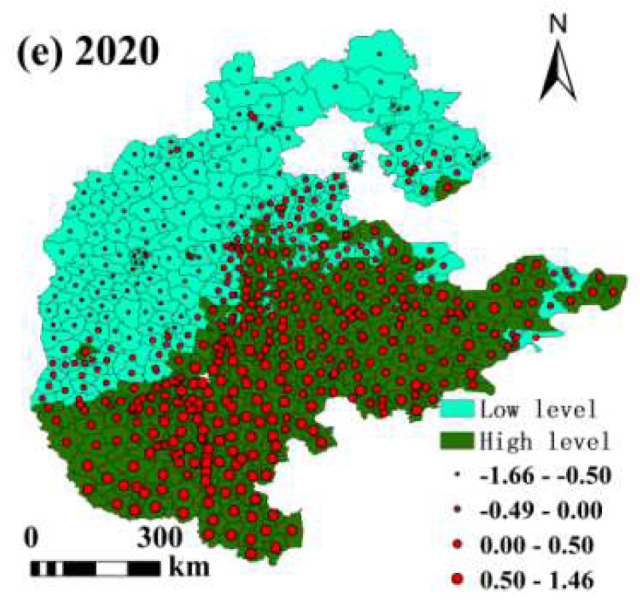
Food security levels from the perspective of food production capacity.

The spatial visualization of food security levels under the perspective of food production capacity and the results of the visualization are shown in Figure 5, which shows that areas with relatively high food production capacity from 2000 to 2020 are mainly concentrated in central, eastern, and southern North China, and these counties have a high food production capacity and correspondingly high levels of food security.

#### 3.5.3. Food Security Assessment Based on Per Capita Food Availability and Food Production Capacity

Based on the classification criteria for per capita food supply, and based on the production capacity of grain, the evaluation level of food security in North China is established, which is divided into three levels: <300 kg/person/year for the food shortage area, 300–400 kg/person/year for the supply–demand balance area, and >400 kg/person/year for the surplus food area. Assuming that P represents per capita food supply and F represents the combined score of food production capacity, the classification criteria based on per capita food supply and the combined food security classification criteria based on food production capacity are shown in Table 5.

In grain-scarce areas, the number of counties with high grain production capacity is increasing year by year, while the number of counties with low grain production capacity is decreasing year by year. The number of counties with high grain production capacity is less than that with low grain production capacity. From the perspective of grain production capacity, it is not difficult to find that in grain-scarce areas, there is hope for an improvement in the situation of food security. In the supply–demand balance zone, the number of counties with low grain production capacity was higher than that of counties with high grain production capacity in 2010 and 2015, and was significantly lower than that of counties with high grain production capacity in other years. The number of counties with high grain production capacity decreased first and then increased, while the number of counties with low grain production capacity increased first and then decreased. The decrease in the number of counties with high grain production capacity was greater than that of counties with low grain production capacity, indicating that the overall grain production capacity of the supply–demand balance zone has declined and there is a certain food security risk. In the surplus grain areas, the number of counties with low grain production capacity is increasing year by year, while the number of counties with high grain production capacity first increases and then decreases. However, the number of counties with low grain production capacity is significantly lower than that of those with high grain production capacity, indicating that most surplus grain areas have good grain production and low food security risks. At the same time, there are also a few regions with lower grain production capacity than the average level of the entire region, indicating potential risks to food security (Table 6 and Table 7).

**Figure 6:**
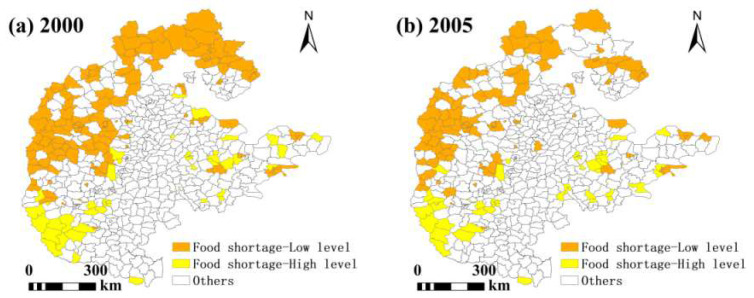

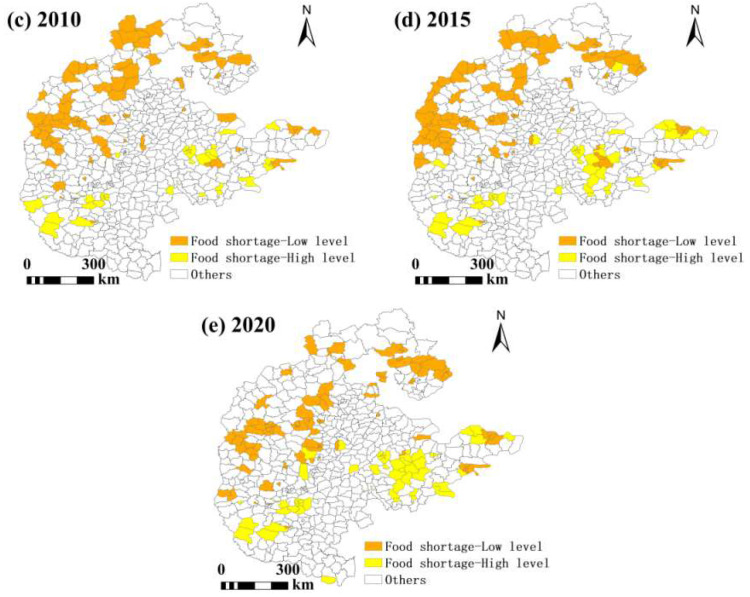
Spatial pattern of food security levels in areas with food shortages.

Spatial visualisation of food security levels in North China shows that in the food shortage area (Figure 6), counties with a low food production capacity are mainly distributed in the northwest and north, with the number decreasing year by year; these counties are not only food shortage areas, but also have low food production capacity, low per capita food supply, as well as food security risks, but food insecurity is difficult to improve by means of agricultural production, and external supply is needed. The counties with a high food production capacity are mainly located in the southwest and the east, and their number has increased over time, with a decrease in the southwest and an increase in the east; these counties have low per capita food availability but have a high potential for development.

**Figure 7:**
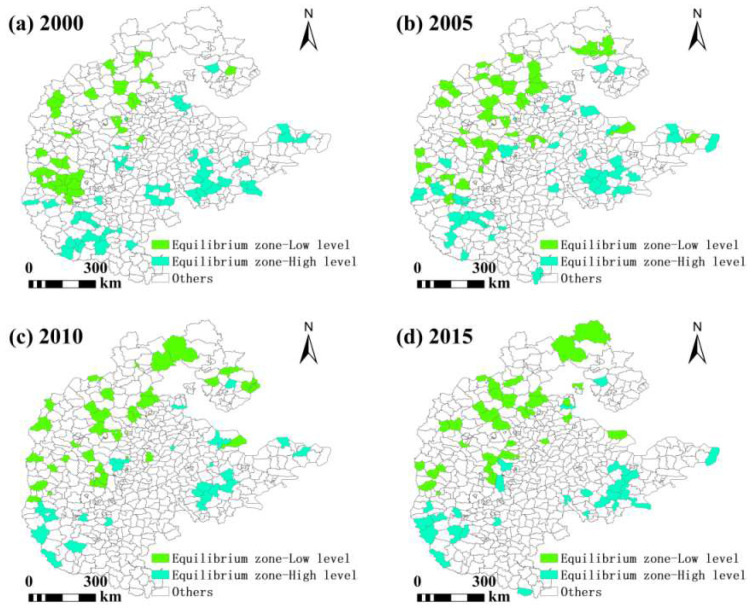

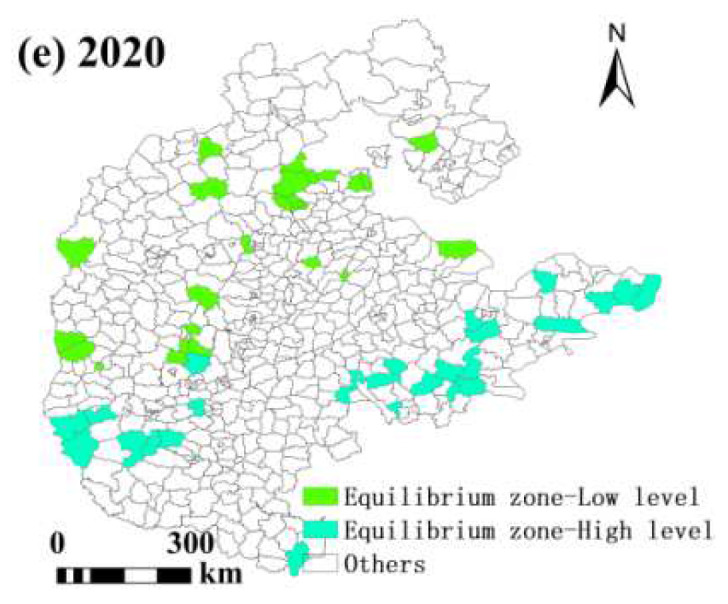
Spatial patterns of food security in zones of equilibrium of supply and demand.

In the zone of balanced supply and demand (Figure 7), counties with a low food production capacity are scattered in the northwest, with a smaller number and decreasing year by year, and the per capita supply in these counties has increased, but the level of food security in terms of food production capacity still needs to be improved. The counties with a high food production capacity are scattered in the southwest and east, with a smaller number and decreasing year by year, and the per capita supply of food in these counties has already increased. These counties have met the minimum standards for food security and have a relatively high food production capacity, so food security is more stable.

**Figure 8:**
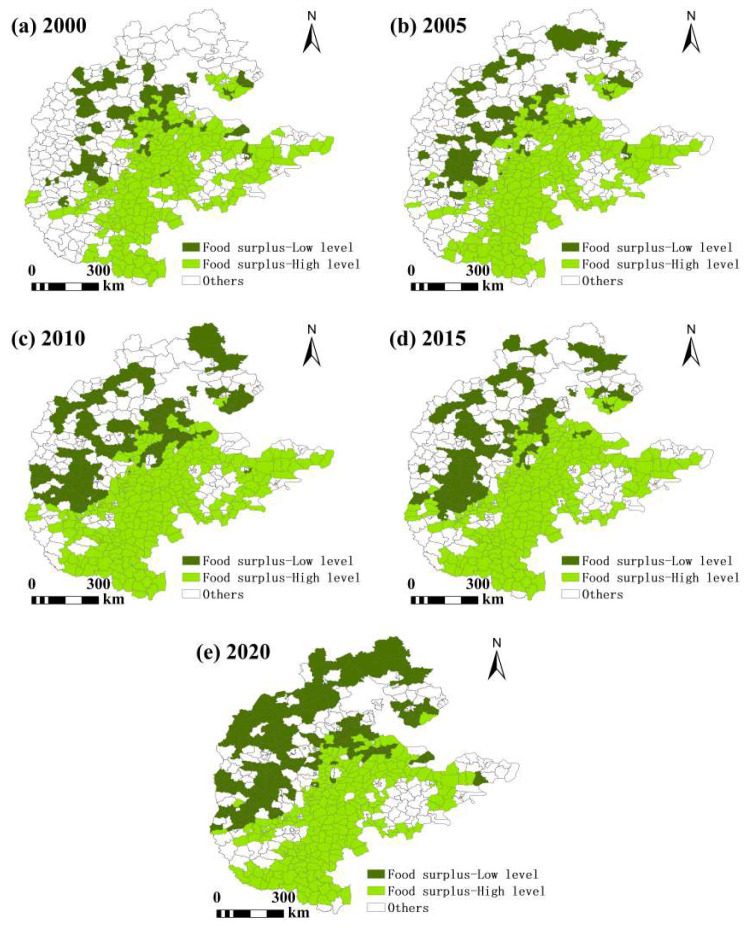
Spatial pattern of food security in regions with grain surplus.

In the food surplus zone (Figure 8), counties with a low food production capacity are mainly located in the northwest, and their numbers are increasing year by year. Although the per capita food supply in these counties has reached the safe standard, food security is still potentially risky from the perspective of the food production capacity. The counties with a high food production capacity are mainly concentrated in the east and the southeast, and their numbers are decreasing over time, but they still occupy an absolute dominant position; these counties are not only surplus food areas, but also have higher levels of agricultural production and higher levels of food security.

## 4. Discussion

### 4.1. Policy Implications

Food security is crucial to national development, and China’s food production and food consumption are among the highest in the world. At present, although the level of food security in China has been greatly ensured, the problems that exist cannot be ignored. For a long time, the government attempted to increase food production as agricultural production as a primary task to grasp, through technical, economic, policy, and other initiatives to vigorously promote food production, so that food production has been greatly improved. However, just increasing food production is not only a fundamental solution for the contradiction between the supply and demand for food, but also brings up a series of resource and environmental pollution problems. At the same time, the domestic and foreign background and environment associated with China’s food security has changed or is changing, resulting in the country’s food security facing new problems, the existing food policy being challenged, and climatic factors and human intervention being the two main factors affecting food production. Based on the conclusions of the study, combined with the current situation of food production in North China, putting forward the corresponding countermeasures to ensure national food security is of great practical significance. In order to ensure national food security and meet the challenges of achieving the goal of “zero hunger” by 2030, this paper draws policy implications in the following areas:

- (1) Give full play to regional advantages, consolidate and enhance the status of food production, and guarantee national food security. The results of the previous study show that there are obvious comparative advantages and agglomeration characteristics of food production in North China, and in the future, we should give full play to the comparative advantages of resources, strengthen the construction of agricultural infrastructure in the high-agglomeration areas, analyse the constraints that exist in the low-agglomeration areas, and adjust the planting structure so as to enable the regions to achieve complementary resources and coordinated development to increase the supply of food and to jointly safeguard food security.
- (2) Improve the disaster early warning capacity, enhance disaster-resistant efficiency, and reduce the impact of climate change on food production. This study verifies that climate has a great impact on food production, that the various influencing factors have caused significant impacts on food production in North China at different times, and that the impacts of extreme climate on food production are more complex than those of conventional climate indicators. Climate change has caused serious impacts on many areas of global natural ecosystems and social production, and agricultural production is more sensitive to climate change and is one of the areas most directly affected by climate change [27,28] and crop yields will be profoundly affected by climate change responses [29]. The impacts of climate change on food production are bidirectional, with changes in temperature altering the temperature conditions for food production, and rising temperatures triggered by increasing atmospheric carbon dioxide shortening the growth cycle of crops and affecting food yields [30], posing a threat to the availability and utilisation of food. Climate change will lead to abnormal precipitation, which will not guarantee the water conditions needed for crop growth, thus causing a reduction in food production [31]. Climate change will also alter the prevalence of pests and increase the frequency of impact pest events, putting agricultural systems at greater risk in the 21st century [32]. The frequency of climate extremes due to climate change can also result in the loss of agro–climatic resources, which in turn affects inter-annual fluctuations in arable land output, and food security risks will be exacerbated by the interplay of other factors [33]. Because different regions face different climatic conditions, the impact of climate on food production in different regions is not the same, and different regions should start from local climatic conditions, formulate relevant policies, and seek food cultivation laws suitable for the region according to local conditions. Therefore, it is urgent to build a climate disaster warning system, actively seek proactive and scientific disaster warning mechanisms, strengthen the protection of corresponding crops in designated areas and key links, achieve precise disaster resistance, and improve disaster resistance efficiency. Among the various meteorological disasters, the area affected by drought is the largest, followed by floods and hail, and the degree of damage varies in different regions, with North China being the most severe [34]. The study in this paper also responds well to this conclusion, so it is more important to improve the accuracy of agrometeorological disaster forecasting, carry out artificial intervention in a timely manner, try our best to reduce the losses caused by climatic disasters, and continuously improve the programme and technology of artificial rain augmentation and other operations so as to reduce the negative impact of climate change on food production.
- (3) Deepen the implementation of the policy of “storing grain in the land”, increasing the protection of arable land and ensuring the area sown with grain. The area of sown grain has a significant effect on grain production in the whole region, so it is necessary to increase the protection of arable land and ensure that the area of sown grain is the most effective means of increasing grain production. In the past 20 years, due to the reclamation of arable land, urban expansion encroachment of arable land, and the implementation of the project of returning farmland to forests and grasslands and a series of other impacts, resulting in China’s arable land in the area size, spatial pattern and quality have produced a great change [35,36,37]. Grain production has a strong dependence on grain sowing area; at present, under the rapid development of urbanisation and high economic growth, the continuous reduction of arable land area in the future is unavoidable, and the difficulty of arable land protection increases. On the basis of an accurate understanding of the impact of the loss of the main arable land on grain production and reasonable control of the transfer of arable land, the continuous development, enhancement, updating, and restoration of the arable land productivity is necessary to safeguard the food supply, an important way to ensure national food security [38]. The government should strictly abide by the red line of farmland protection, plan reasonably, and focus on controlling the encroachment of various types of construction land on farmland. It should curb the trend of non-grain and non-agricultural use, and at the same time, timely compensate for the quantity of farmland to ensure the quantity, quality, and production capacity of farmland; monitor the quality of cultivated land, with a focus on monitoring its ability to maintain soil and water conservation, soil pollution, and soil organic matter content, to ensure the quality of cultivated land; level farmland and improve farmland conditions to enhance the efficiency of grain production; and minimize the occupation of high-quality farmland as much as possible, stabilizing the planting area of grain, and ensuring food security.
- (4) Resolutely implement the strategy of “storing grain in technology” and increase investment in agricultural science and technology. The total power of agricultural machinery significantly promoted grain production in North China from 2000 to 2020. The increase in grain production is closely related to the progress of modern agricultural technology, especially agricultural machinery. Since the reform and opening up of agricultural technology, the total power of agricultural machinery in China has shown a continuous upward trend, and the level of socialized operation of agricultural machinery has continued to improve, effectively promoting labour production efficiency and land production efficiency [39]. Agricultural mechanisation can significantly promote grain yield increase [40]. Improving the total power of agricultural machinery can effectively increase grain production [41]. This study also indicates that the total power of agricultural machinery has a significant promoting effect on grain production in North China. Land still has bottlenecks in increasing grain production, but technology has enormous potential in promoting grain production. The level of agricultural mechanisation used to be an important factor in promoting the increase of grain production in China. Although China continues to promote the development of agricultural mechanisation, the overall level of agricultural mechanisation in China is still not high, and there is still a lot of room for improvement. With the help of agricultural mechanisation, there is a high possibility of further increasing grain production, and agricultural mechanisation is the only way to achieve agricultural modernisation. Therefore, in order to improve China’s ability to ensure national food security, it is necessary to further promote the use of agricultural mechanisation.
- (5) Improve the efficiency of fertiliser use. The application of chemical fertilisers over the years has a significant promoting effect on grain production. The use of fertilisers not only increases grain production but also brings serious environmental pollution problems. In view of this, China began implementing large-scale soil testing and formula fertilisation subsidy projects in 2005. Soil testing and formula fertilisation technology have shown unique advantages in reducing fertiliser application, improving fertiliser utilisation efficiency, and protecting the environment [42,43]. At present, some county-level farmers have small production scales and relatively backward production technologies, making policy implementation difficult. It is recommended that the government increase investment in soil testing and formula fertilisation technology, strengthen the promotion and implementation of soil testing and formula fertilisation technology, increase investment in technology, develop clean fertilisers such as organic fertilisers to replace traditional inorganic fertilisers, and mitigate pollution caused by fertiliser use. Appropriate measures can be taken to reduce the planting area of crops that consume a large amount of fertiliser, improve the efficiency of fertiliser use, and reduce soil nutrient loss and environmental pollution caused by returning straw to the field and increasing organic fertiliser. It is also important to change agricultural production methods; adopt crop rotation and other systems to ensure soil fertility and improve grain production capacity starting from the actual situation of each county, formulate pollution prevention and control policies suitable for local fertiliser use; introduce laws and regulations related to fertiliser use; continuously regulate the use of fertilisers by farmers; effectively control the amount of fertiliser used; improve the efficiency of fertiliser use; and ensure the sustainable development of agriculture.

### 4.2. Limitations

Focusing on food security in North China, this study specifies the research scale to the county level and quantitatively assesses the impacts of climate change and human interventions on food production and food security in North China in order to refer to the practical experience and discuss adaptive countermeasures to increase food production and reduce the food security risk while maintaining the agricultural system. Although corresponding research results have been achieved, due to the limitations of information acquisition and data sources, some research-worthy issues have still not been explored in depth, and the study still has some deficiencies that need to be further explored and resolved in future research, such as the following:

- (1) This study of grain production in North China, due to the limitations of resources, time, statistics, and other factors, is only a study of the overall grain, but not the specific grain varieties; there are certain flaws and shortcomings. In future research, the grain structure should be subdivided for wheat, corn, and other different types of grain research. Research on the production of different varieties of grain and factors affecting the production of grain production in North China counties can be more targeted to give the relevant policies and recommendations.
- (2) There are many other factors that affect food production and food security. In this study, only some of the climatic and anthropogenic factors were selected, but in fact, other climatic factors (e.g., winter frost, inversion, carbon dioxide concentration, etc.) and anthropogenic factors (e.g., storage of food, greenhouse gas emissions, soil pollution, irrigation, policies, etc.) have a greater impact on food production in the process of growing food crops. In future studies, it is necessary to select as many factors as possible that have an impact on food production to improve the reliability of the results.

## 5. Conclusions

- (1) Grain production in northern China is on an upward trend, with 2003 marking a turning point in grain production. Grain production hot spots are concentrated in Henan and Shandong.
- (2) Climate change and human activities together caused changes in food production in northern China, with climate change contributing 6.52% to food production and human activities contributing 93.48%, indicating that human activities had a greater impact on changes in food production in northern China.
- (3) There are obvious spatial differences in food security in northern China. As time progresses, the area of food-shortage zones decreases in size, becoming evenly distributed and dispersed from the initial concentration in northern Hebei and most of Shanxi. The supply–demand balance zone is not clearly characterised by changes. The area of food surplus zones increases in size, with a tendency to expand from the south and centre of the country to the west and north. Counties in the northwest and north generally have a low food production capacity and are potentially at risk of food insecurity, even if they are located in surplus food areas, while in the east and south, the per capita food supply situation is likely to be mitigated because of the high food production capacity, even if they are located in food deficit areas.

## Figures and Tables

**Table 1:** Results of LM and R-LM tests.

Year	Test	LMlag	R-LMlag	LMerr	R-LMerr
2000	statistical value	65.5542 ***	52.3269 ***	341.1832 ***	327.9559 ***
	*p*-value	0.000	0.000	0.000	0.000
2005	statistical value	43.7521 ***	36.1590 ***	157.2363 ***	149.6432 ***
	*p*-value	0.000	0.000	0.000	0.000
2010	statistical value	22.6522 ***	18.7132 ***	54.4440 ***	50.5050 ***
	*p*-value	0.000	0.000	0.000	0.000
2015	statistical value	37.0146 ***	33.1430 ***	48.1459 ***	44.2743 ***
	*p*-value	0.000	0.000	0.000	0.000
2020	statistical value	3.0194 *	2.8053 *	1.7888	1.5748
	*p*-value	0.082	0.094	0.181	0.210

Note: *, *** represent passing the significance test of 0.1, and 0.01.

**Table 2:** Spatial econometric model results.

Variable	2000	2005	2010	2015	2020
	Coefficients and *p*-Values	Coefficients and *p*-Values	Coefficients and *p*-Values	Coefficients and *p*-Values	Coefficients and *p*-Values
lnTem	−0.827 (0.154)	0.969 (0.180)	−0.741 (0.560)	1.057 (0.158)	0.342 (0.703)
lnPre	0.782 (0.418)	2.781 (0.106)	−268 (0.654)	0.170 (0.820)	0.296 (0.719)
lnTem3-10	0.596 (0.474)	−0.235 (0.856)	3.891 * (0.064)	−0.763 (0.340)	0.480 (0.719)
lnPre3-10	−0.637 (0.499)	−2.419 (0.160)	1.701 (0.542)	−0.009 (0.989)	−0.120 (0.879)
lnSsd	0.802 ** (0.047)	0.493 (0.110)	−6.405 (0.252)	−0.278 (0.406)	−0.117 (0.633)
lnTa ≥ 0	3.208 (0.272)	−1.447 (0.678)	4.078 (0.252)	−10.233 *** (0.000)	2.532 (0.459)
lnTa ≥ 10	−0.464 (0.553)	0.311 (0.914)	−6.405 ** (0.031)	7.862 *** (0.001)	−0.686 (0.510)
lnCdd	−0.081 (0.508)	0.449 ** (0.019)	−0.256 * (0.087)	0.031 (0.811)	0.598 * (0.023)
lnGsl	−0.485 (0.490)	−0.794 (0.524)	−0.176 (0.804)	1.622 ** (0.027)	−1.398 * (0.097)
lnDtr	−1.345 *** (0.000)	−0.393 (0.225)	0.488 (0.195)	−0.259 (0.434)	0.549 * (0.097)
lnPop	0.020 (0.720)	−0.012 (0.841)	0.011 (0.868)	0.005 (0.937)	0.130 ** (0.022)
lnGdp	0.071 ** (0.037)	−0.034 (0.286)	−0.101 ** (0.005)	0.068 * (0.062)	−0.078 ** (0.042)
lnGsa	0.608 *** (0.000)	0.910 *** (0.000)	0.256 *** (0.000)	0.830 *** (0.000)	0.838 *** (0.000)
lnFer	0.369 *** (0.000)	0.157 *** (0.000)	0.400 *** (0.000)	0.194 *** (0.000)	0.217 *** (0.000)
lnMac	0.100 *** (0.000)	0.091 ** (0.028)	0.383 *** (0.000)	0.137 *** (0.006)	0.052 *** (0.000)
lnElec	−0.013 (0.550)	0.057 ** (0.010)	0.007 (0.783)	−0.020 (0.384)	−0.043 * (0.063)

Note: *, **, *** represent passing the significance test of 0.1, 0.05, and 0.01.

**Table 3:** Contribution of climate change and human activities to changes in grain production in North China.

	Factors	Importance of Characteristics (%)					
		2000	2005	2010	2015	2020	Average Value
Climate factors	Tem	0.40	0.40	0.60	0.40	0.30	0.42
	Pre	0.50	0.40	0.50	0.80	0.60	0.56
	Tem3-10	0.60	0.40	0.80	0.60	0.30	0.54
	Pre3-10	0.50	0.30	0.50	0.60	0.40	0.46
	Ssd	1.10	0.90	1.10	1.40	0.80	1.06
	Ta ≥ 0	0.40	0.30	0.80	0.50	0.40	0.48
	Ta ≥ 10	0.50	0.50	0.80	0.70	0.30	0.56
	Cdd	0.90	0.90	0.90	0.90	1.10	0.94
	Gsl	0.70	0.90	1.00	0.70	0.40	0.74
	Dtr	0.80	0.80	1.10	0.60	0.50	0.76
Human activity factors	Pop	1.90	1.60	1.30	0.80	1.30	1.38
	GDP	0.80	0.90	0.70	0.60	0.90	0.78
	Gsa	23.50	80.70	69.80	85.60	88.50	69.62
	Fer	58.40	5.40	9.00	2.10	1.40	15.26
	Mac	8.50	3.70	9.60	2.70	1.80	5.26
	Elec	0.70	2.00	1.60	1.10	0.90	1.26

**Table 4:** Food security evaluation indicators.

Basic Conditions	X1	Grain Production
Climatic conditions	X2	Tem
	X3	Pre
	X4	Tem3-10
	X5	Pre3-10
	X6	Ssd
	X7	Ta ≥ 0
	X8	Ta ≥ 10
	X9	Cdd
	X10	Gsl
	X11	Dtr
Human activity	X12	Pop
	X13	GDP
	X14	Gsa
	X15	Fer
	X16	Mac
	X17	Elec

**Table 5:** Criteria for food security classification.

Partition Type	Delineation Criteria	
	P	F
Food shortage—low level	<300	<0
Food shortage—high level	<300	>0
Equilibrium zone—low level	300–400	<0
Equilibrium zone—high level	300–400	>0
Residual food—low level	>400	<0
Food surplus—high level	>400	>0

**Table 6:** Number of counties with different types of food security.

Type	Number of Counties				
	2000	2005	2010	2015	2020
Food shortage—low level	131	104	84	104	96
Food shortage—high level	47	48	38	46	54
Equilibrium zone—low level	33	38	36	32	25
Equilibrium zone—high level	50	52	25	27	26
Residual food—low level	80	96	141	114	144
Food surplus—high level	236	239	253	254	231

**Table 7:** Percentage of food production capacity in different per capita food supply zones.

Categorisation	Grain Production Capacity	Number of Counties				
		2000	2005	2010	2015	2020
Food shortage areas	Low level	73.60%	68.42%	68.85%	69.33%	64.00%
	High level	26.40%	31.58%	31.15%	30.67%	36.00%
Equilibrium zone	Low level	39.76%	42.22%	59.02%	54.24%	49.02%
	High level	60.24%	57.78%	40.98%	45.76%	50.98%
Food surplus areas	Low level	25.32%	28.66%	35.79%	30.98%	38.40%
	High level	74.68%	71.34%	64.21%	69.02%	61.60%

## Data Availability

The original contributions presented in the study are included in the article, further inquiries can be directed to the corresponding author.
